# A case of aggressive aortic prosthetic valve endocarditis aggressive caused by *Staphylococcus lugdunensis*

**DOI:** 10.1186/s40792-020-01062-x

**Published:** 2020-11-05

**Authors:** Kazuhiro Yamazaki, Kenji Minakata, Kazuhisa Sakamoto, Jiro Sakai, Yujiro Ide, Masahide Kawatou, Hideo Kanemitsu, Tadashi Ikeda, Kenji Minatoya, Ryuzo Sakata

**Affiliations:** Department of Cardiovascular Surgery, Graduate of School of Medicine, Kyoto University, 54 Shogoin-Kawahara-Machi, Sakyo, Kyoto, Japan

**Keywords:** *Staphylococcus lugdunensis*, Prosthetic valve endocarditis, Aortic valve replacement

## Abstract

**Background:**

*Staphylococcus lugdunensis* is a coagulase-negative *Staphylococcus* species, which are weak pathogenic bacteria generally. However, the acute and severe pathogenicity of *Staphylococcus lugdunensis* infective endocarditis may be due to the rapid growth of large vegetation and consequent valve destruction.

**Case presentation:**

The patient was an 81-year-old male who visited our hospital with chief complaints of low back pain and high fever. Four years before this visit, he had undergone aortic valve replacement for aortic regurgitation. He was found to be hypotensive. Although there is no heart murmur on auscultation and echocardiography revealed negative findings with aortic valve, a blood test showed increases in the white blood cell count and C-reactive protein concentration. On the next day, Gram-positive cocci were detected in a blood culture and echocardiography detected a large vegetation on the prosthetic valve with increased flow velocity. Therefore, he underwent redo aortic valve replacement emergently. *Staphylococcus lugdunensis* was identified in blood samples and vegetation culture. Consequently, the patient was treated with antibiotics for 5 weeks after the operation and discharged home.

**Conclusions:**

We experienced rapidly progressive prosthetic valve endocarditis caused by *Staphylococcus lugdunensis*. Hence, *Staphylococcus lugdunensis* infective endocarditis requires aggressive treatment, and the pathogenicity of this coagulase-negative Staphylococcus with high drug susceptibility should not be underestimated.

## Background

*Staphylococcus lugdunensis* (*S. lugdunensis*) is a coagulase-negative *Staphylococcus* species (CNS; i.e., gram-positive cocci). Generally, CNS are weak pathogenic bacteria that colonize the skin. However, diseases associated with *S. lugdunensis*, particularly infective endocarditis (IE), are acute, may cause valve destruction, and often follow a lethal course. In this study, we report a case of a patient who developed an acute prosthetic valve endocarditis (PVE) caused by *S. lugdunensis* 4 years after an aortic prosthetic valve replacement.

## Case presentation

**Fig. 1:**
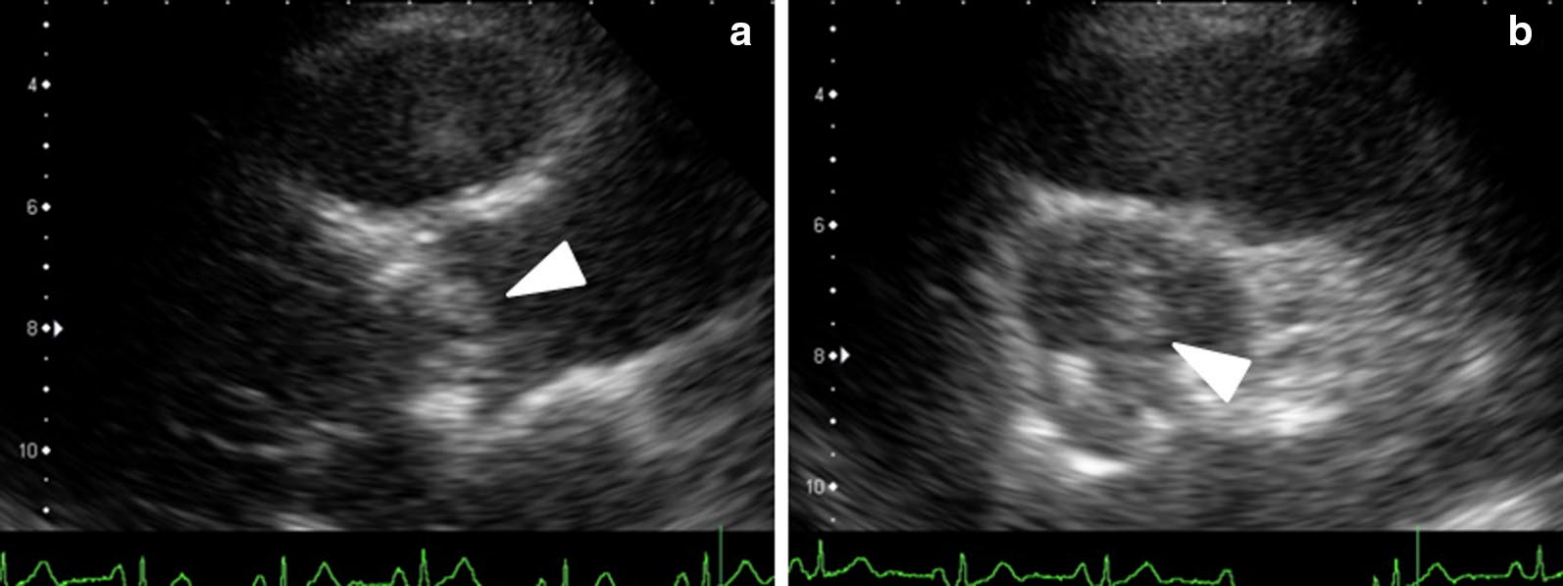
**a** 2D echocardiogram. Apical long-axis view shows a large vegetation (arrow), measuring 16 mm × 9 mm. **b** Short-axis view

The patient was an 81-year-old male who visited our hospital with chief complaints of low back pain and fever. Four years before, he had undergone AVR with a 23-mm Carpentier-Edwards Perimount (CEP) valve (Edwards Lifesciences, Irvine, CA, USA) to treat aortic regurgitation (AR). He had developed low back pain at 14 days and fever at 10 days before admission to our hospital. Because the symptoms persisted even after medical treatment at a local physician, he visited the Department of Cardiology of our hospital. The following physical findings were observed during his visit: body temperature, 39.0 °C; blood pressure, 94/58 mmHg; pulse rate, 90/min; and no heart murmur on auscultation. A blood test revealed increases in the white blood cell count and C-reactive protein, and a mild increase in the hepatobiliary enzyme concentrations. Although computed tomography (CT) was immediately performed, no abscess formation or pneumonia was detected. Similarly, echocardiography revealed a trivial degree of mitral regurgitation, and negative findings for AR and tricuspid regurgitation. Because of his high fever and AVR history, he was emergently admitted to our hospital and treated with cefazolin (CEZ). Moreover, a continuous norepinephrine infusion was needed for the hypotension. On the next day, *Staphylococcus* bacteria were identified by blood culture tests performed on admission. Accordingly, teicoplanin (TEIC) was added along with CEZ. The second echocardiography evaluations detected a large vegetation measuring 16 mm × 9 mm in diameter (Fig. 1) on the prosthetic valve with increased transvalvular gradient (peak velocity, 4.4 m/s; peak pressure gradient, 79 mmHg), leading to a staphylococcal PVE diagnosis. Therefore, the patient was referred to our department for emergency surgery.

### Surgical procedures

**Fig. 2:**
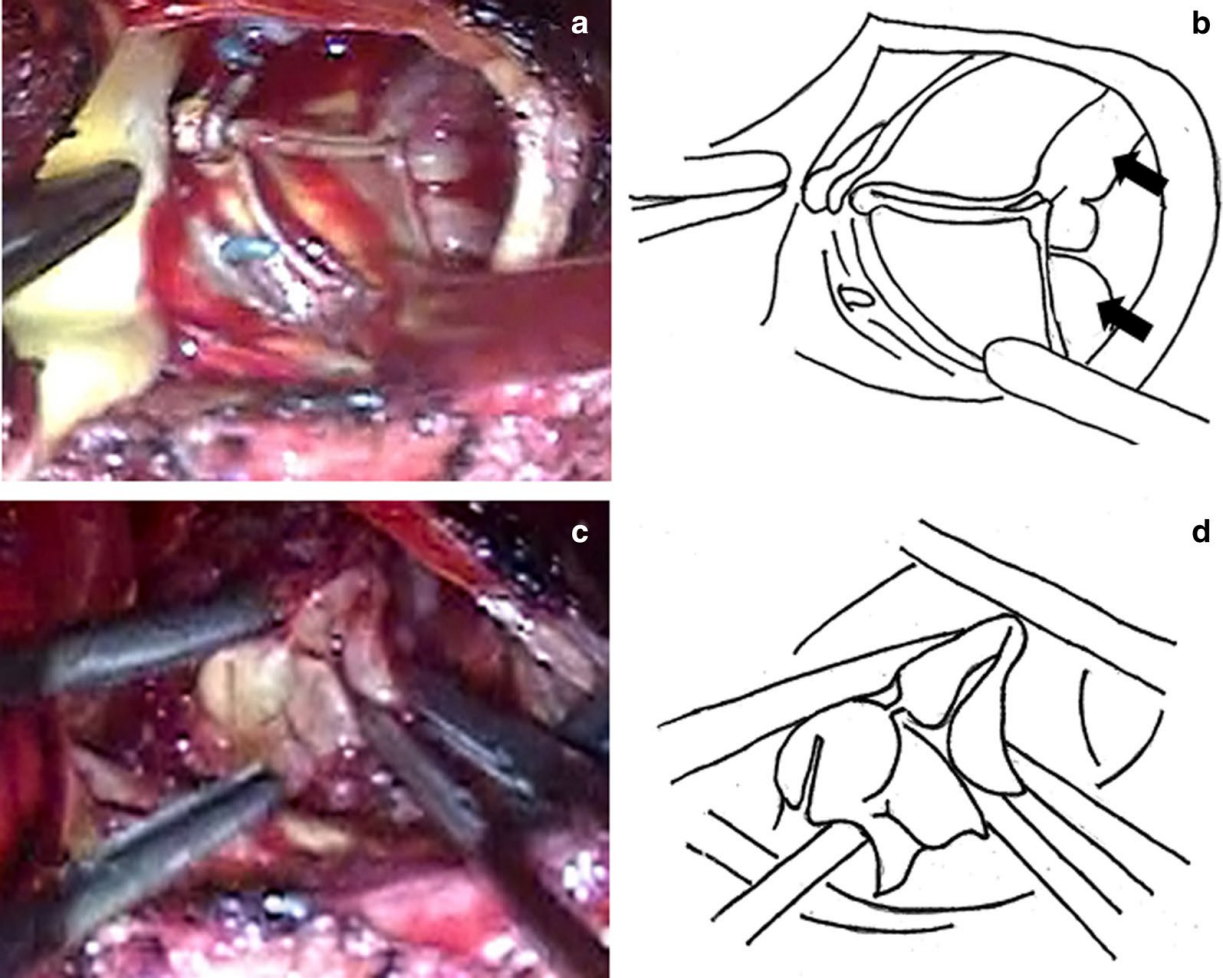
**a**, **b** The prosthetic valve covered with vegetation (arrow). **c**, **d** Large vegetation covers the outflow tract

**Fig. 3:**
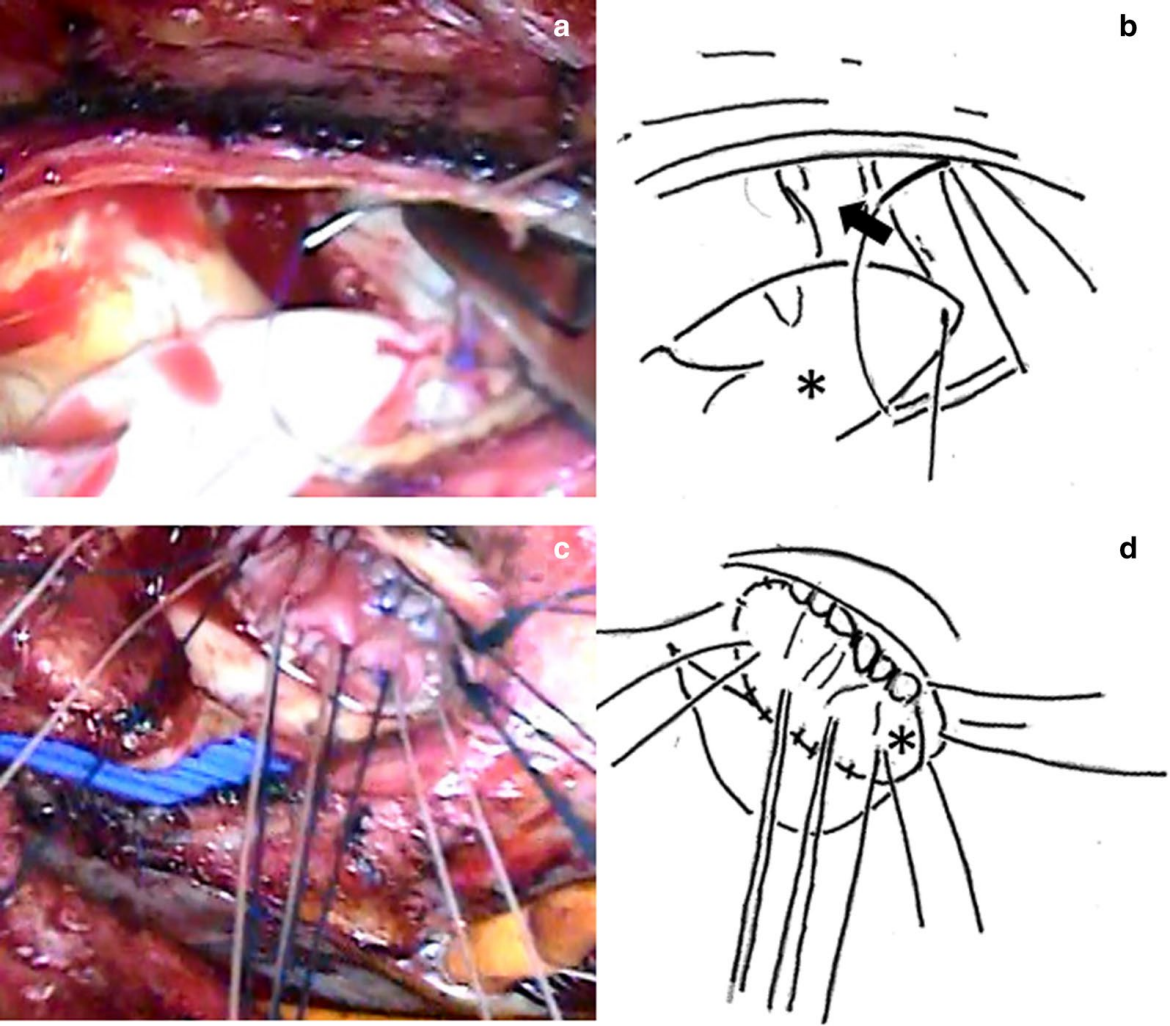
**a**, **b** After copious irrigation of the abscess cavity (arrow), an aortic annulus reconstruction was carried out with a bovine pericardium patch (asterisk) using continuous 4-0 monofilament sutures. **c**, **d** Suturing from outside of the aortic root to the bovine aortic annulus (asterisk) with non-everting mattress

Redo sternotomy was performed, and a cardiopulmonary bypass (CPB) was established. Then, an antegrade-cardioplegia was delivered after aortic cross-clamping, and cardiac arrest was quickly achieved. A transverse incision was performed just above the previous incision on the proximal ascending aorta to expose the prosthetic valve, which was covered with vegetation (Fig. 2a, b). No defects were observed in the valve seating, which was covered with endothelial tissue, when viewed from the aortic side. Subsequently, the prosthetic valve, which had been implanted in the intra-annular position, was removed by cutting the sutures. It should be noted that pus leakage was observed from the commissure and annulus between the left coronary and noncoronary sinuses to the nadir of the two cusps. Therefore, the patient was diagnosed with aortic annular abscess. After complete prosthetic valve removal, large vegetation covering the left ventricular outflow tract was revealed (Fig. 2c, d). A visual inspection after aortic annular abscess removal demonstrated that the infection was limited to the annulus. Moreover, no further infection was detected between the left ventricular outflow tract and the mitral valve annulus. After copious irrigation of the abscess cavity, an aortic annulus reconstruction was carried out with a bovine pericardium patch using continuous 4-0 monofilament sutures (Fig. 3a, b). A new aortic annulus was sized to a 23-mm sizer, but it was slightly tight. Therefore, a 21-mm CEP valve was chosen. The part of the patch between the LCC and NCC was threaded from the outside of the aorta with the patch and sutured with interrupted sutures without a pledget (Fig. 3c, d). In the other parts, we used the everting sutures, and the artificial valve was attached to the intra-annular position. The aortic cross-clamping was released, and spontaneous beating resumed. The patient was smoothly weaned from the CPB.

### Postoperative course

The patient had stable hemodynamics and respiratory function and was extubated on postoperative day 1. Because he was found to have aortic annular abscess, three antibiotics, including gentamicin (GM), TEIC, and CEZ, were administered. In addition, gamma globulin was also given for 3 days postoperatively. At that time, the infecting organism was identified as *S. lugdunensis* by a preoperative blood culture and intraoperative vegetation culture. Due to the high drug susceptibility, the CEZ dose was increased from 3 to 6 g/day, and the antibiotic treatment regimen was changed to CEZ, continuous GM, and oral rifampicin (RFP, 300 mg every 8 h). TEIC administration was stopped. After 4 weeks of postoperative continuous CEZ administration, the antibiotic treatment regimen was changed to oral RFP and sulfamethoxazole–trimethoprim (ST). At 5 weeks postoperatively, the patient was uneventfully discharged home. Outpatient echocardiography and CT follow-ups revealed no abnormalities, and the oral antibiotics were discontinued at 1 year postoperatively. The subsequent clinical course was uneventful. An outpatient follow-up conducted 5 years postoperatively showed no recurrence.

## Discussion

*Staphylococcus lugdunensis* is one of the CNS that was first reported in 1988 [1]. CNS are involved in the formation of biofilms, which can adhere to implanted devices and reduce antibacterial activity. PVE is commonly caused by CNS. The overall IE incidence caused by *S. lugdunensis* is low (0.8%) [2]. However, the high native valve endocarditis (NVE) incidence caused by CNS is a characteristic feature. For example, the NVE, PVE, and pacemaker infection incidences caused by CNS are approximately 80%, 10%, and 10%, respectively [2, 3]. According to a literature review by the American Society for Microbiology, NVE caused by *S. lugdunensis* accounts for 44% of all NVE cases caused by CNS [4].

In addition, most CNS are reported to have low pathogenicity levels and colonizes the skin and mucous membranes of humans. However, a recent report of a highly invasive infection caused by *S. lugdunensis* demonstrates the high pathogenicity of this bacterial species. According to other reports, the mortality rate associated with IE caused by *S. lugdunensis* (38–50%) [2, 3] is higher than that associated with IE caused by *S. aureus* (7–27%) [2, 5]. Particularly, PVE mortality caused by *S. lugdunensis* is very high (78%) [3].

The acute and severe pathogenicity of *S. lugdunensis* IE, unlike typical IE caused by CNS, may be due to the rapid growth of large vegetation and consequent valve destruction. In many previous studies, preoperative tests identified large vegetation that had quickly formed after the emergence of symptoms. Other intraoperative findings such as leaflet perforation and aortic annular abscess have also been reported [6–10]. In the present case, during his visit our hospital, there is no heart murmur on auscultation and echocardiography revealed negative findings with aortic valve. However, on the next day, echocardiography detected a large vegetation on the prosthetic valve with increased flow velocity. Therefore, our findings are consistent with those of previous studies that demonstrated a very rapid vegetation progression. Regarding the intraoperative findings, we observed large vegetation that covered the entire prosthetic valve and aortic annular abscess. These features could have contributed to systemic embolism, heart failure, and even increased mortality if not treated in a timely fashion. Consequently, this case suggests that IE caused by *S. lugdunensis* may cause very rapid progression and requires emergency surgery.

## Conclusion

In this report, we described a case of acute PVE caused by *S. lugdunensis* that was immediately treated with a good outcome. Despite the low incidence, *S. lugdunensis* IE requires aggressive treatment, and the pathogenicity of this CNS with high drug susceptibility should not be underestimated.

## Data Availability

Data sharing is not applicable to this article, as no datasets were generated or analyzed during the study.

## Abbreviations

*S. lugdunensis*: *Staphylococcus lugdunensis*
CNS: Coagulase-negative *Staphylococcus* species
IE: Infective endocarditis
PVE: Prosthetic valve endocarditis
CEP: Carpentier-Edwards Perimount
CT: Computed tomography
CEZ: Cefazolin
TEIC: Teicoplanin
CPB: Cardiopulmonary bypass
GM: Gentamicin
RFP: Rifampicin
ST: Sulfamethoxazole–trimethoprim
NVE: Native valve endocarditis
